# A Non-Laboratory Gait Dataset of Full Body Kinematics and Egocentric Vision

**DOI:** 10.1038/s41597-023-01932-7

**Published:** 2023-01-12

**Authors:** Abhishek Sharma, Vijeth Rai, Melissa Calvert, Zhongyi Dai, Zhenghao Guo, David Boe, Eric Rombokas

**Affiliations:** 1 Mechanical Engineering, University of Washington, Seattle, 98195 USA; 2 Electrical and Computer Engineering, University of Washington, Seattle, 98195 USA; 3 Electrical Engineering and Computer Science, Massachusetts Institute of Technology, Cambridge, 02142 USA

**Keywords:** Machine learning, Public health, Mechanical engineering, Scientific data, Biomedical engineering

## Abstract

In this manuscript, we describe a unique dataset of human locomotion captured in a variety of out-of-the-laboratory environments captured using Inertial Measurement Unit (IMU) based wearable motion capture. The data contain full-body kinematics for walking, with and without stops, stair ambulation, obstacle course navigation, dynamic movements intended to test agility, and negotiating common obstacles in public spaces such as chairs. The dataset contains 24.2 total hours of movement data from a college student population with an approximately equal split of males to females. In addition, for one of the activities, we captured the egocentric field of view and gaze of the subjects using an eye tracker. Finally, we provide some examples of applications using the dataset and discuss how it might open possibilities for new studies in human gait analysis.

## Background & Summary

Gait analysis is a fundamental tool that is used broadly. Motion capture can be used for clinical gait analysis, with real implications for prescription of interventions, functional classification, and prescription of assistive devices. For basic science, motion capture provides a window into how the brain generates movement, captures sensation, and how neural operations interact with the biomechanics of the body.

Practical considerations have generally restricted gait analysis to within-laboratory experiments. Motion capture has required controlled conditions and apparatus, and this has resulted in relatively controlled and restricted movement datasets. In order to make justifiable comparisons, we have generally narrowed the scope of movement analysis into that which was reasonably measurable given the technology of the time. This has proven fruitful. We now understand a great deal about movement in laboratory conditions. What is less clear is how different movement is outside of those conditions. Are movements generated by people in their daily lives significantly different than those they perform in laboratory experiments?

In other words, those studying movement intuit that outside of laboratory conditions, behavior is likely to be less clean and of greater complexity. The precise nature of this complexity, and a quantitative understanding of it, remains open. In addition, there is evidence that artificial environments can compromise ecological validity. For example, it has been shown^1^ that how participants walk changes by a small but significant amount depending on the number of researchers present during the experiment. With the dataset we present here, we intend to contribute data captured outside of the lab to enable comparative analyses with in-laboratory activities. This kind of data can be used to train data-driven predictive models of human movement^2,3^, or activity^4,5^ or terrain^6^ classification.

In this manuscript we describe a multi-subject full body kinematics dataset that captures diverse movements including forward walking, backward walking, side stepping, avoiding obstacles by stepping over them, navigating around obstacles in controlled environments as well as in uncontrolled and unrestricted natural environments like classrooms, atrium and staircases. For the unrestricted activities, we also present the corresponding egocentric video and gaze data captured using an eyetracker. These data provide a window into the role that the environment plays in determining how we move.

### On the uniqueness of the dataset

Most gait datasets currently available fall under the following categories: (1) Full-body kinematics or kinetics from level-ground walking or running in a lab environment, comprised of straight walking (~10 m) at different self-selected speeds^7–11^, (2) full-body kinematics or kinetics from treadmill walking or running at pre-defined speeds^7,11–13^, (3) standing data collected on perturbation platforms^14,15^, and (4) staircase walking on in-lab staircases with 4–7 steps^11,16^. For a more comprehensive review of stair ambulation, please refer to^17^. These datasets were collected using in-lab motion capture systems and collectively comprise most of the publicly available gait datasets.

Recently, several gait datasets of diverse and more complex activities have been made publicly available^11,18–22^. HuGaDB^18^ is a dataset collected from 18 participants performing several activities. The data include inertial measurement unit (IMU) recordings from sensors strapped to the thigh, shank and feet, and don’t include full body joint kinematics^19^. captured full-body kinematics from different activities in a lab environment. Most of the activities included data from 1–2 subjects (maximum 27 subjects for walking). In that dataset, most subjects performed 1–2 trials (maximum 27 trials from 1 subject, for walking) where each trial consisted of 7–10 seconds of walking^20^. captured full-body kinematics on a ramp, level ground, and stairs. Data were collected from 10 subjects, with 2 trials from each subject. A ramp and an 8-step staircase were replicated inside the lab. The exact dimensions of the course are not specified, but from the provided image of the environment, we estimate that each trial consisted of 60–70 steps^21^. collected data in a natural out-of the lab environment in 3 different terrain types. However, movement captured is mostly straight-line walking, and the data size is relatively small (6 subjects with 5–6 minutes or 500–600 steps of walking data per subject). In this dataset, we present full body kinematics data from multiple activities, with at least 5 minutes of data per subject for each activity.

Usually, human movement datasets focus on capturing several instances of a narrow activity or a specific maneuver. However, humans perform high-level tasks like going into a room or moving towards a goal, and select the lower level maneuvers instinctively, as per the moment to moment requirements. In order to best replicate this kind of behavior, in the dataset we present here, specific paths were not prescribed to subjects. The subjects were only given ‘high-level’ instructions like ‘go into that classroom’ or ‘Walk up that stairwell’ or ‘Walk towards and sit on that chair’. Then, the ‘lower-level’ movements were decided by the subject. We expect this to have captured more natural movements as well as variability in movement strategies.

In this dataset, subjects naturally transition between different classrooms, an atrium, and stairwells. These transitions include opening doors, changing between flat ground and stair walking, and performing sit to stand and stand to sit movements.

In addition, few datasets include full body motion capture along with the egocentric vision and gaze of the subjects. This limits our understanding of how environments affects human gait from moment to moment. We are aware of only one dataset^21^ that captures full body motion capture and egocentric vision data in natural environments. Here, we provide another, in an effort to enable improved understanding of how movement is conditioned on the environment.

## Methods

### Experiments and participants

The dataset is comprised of 6 activities: (1) Level-ground obstacle-free walking, (2) Level-ground walking with random stops, (3) Staircases spanning a 6-storey building, (4) The Comprehensive High-Level Activity Mobility Predictor (CHAMP) activity set, (5) Controlled obstacle course, and (6) Unrestricted walking in public spaces. What we mean by controlled, as well as detailed descriptions of the activities, are presented in the Methods section. Numbers of participants and demographic information for each of the activities is shown in Table 1. The IDs column shows the subject naming convention for the activity i.e. data for a given activity can be found in the directories with the corresponding subject ID.

**Table 1:** Activities and Subject Details.

Activities	Sensory Modalities	IDs	# subjects	Age (yrs)	Height (cm)	Duration
Level-ground obstacle free	Full-body kinematics	xMF*	5 females 6 males	26.2 ± 2.7	174 ± 10.9	2.19 hrs
Level-ground walking with random stops	Full-body kinematics	xMF*	5 females 6 males	26.2 ± 2.7	174 ± 10.9	2.48 hrs
Staircases spanning a 6-storey building	Full-body kinematics	xMF*	5 females 6 males	26.2 ± 2.7	174 ± 10.9	2.11 hrs
Modified CHAMP	Full-body kinematics	xOA*	5 females 5 males	21.5 ± 2.4	173.4 ± 6.9	2.36 hrs
Controlled obstacle course	Full-body kinematics	xOA*	5 females 5 males	21.8 ± 2.2	172.8 ± 6.8	3.16 hrs
Unrestricted walking in public spaces	Full-body kinematics egocentric vision and gaze	xUD*	12 females 11 males	22.8 ± 2.7	171.2 ± 9.7	11.8 hrs

Subjects were recruited through campus email and social media. All activities were approved by the Institutional Review Board at University of Washington (STUDY00004707 “Smart Step”).

### Equipment

Subjects’ joint kinematics and center of mass positions were recorded using an Xsens full body motion capture system^23^, using IMUs, recorded at 60 Hz for all the activities. For activity 6, egocentric vision and gaze data were collected using a binocular eye tracker from Pupil Labs^24^ recorded at 30 Hz.

### Description of activities

As listed in Table 1, we collected data from 6 activities. These activities were recorded in out-of-the lab environments. The environments include a long corridor, staircases spanning 6 floors of building with flat sections the middle, Classrooms, an Atrium. The environments are not controlled, with large number of people using the atria and classrooms in some instances, and several encounters with unexpected people while turning are present in the dataset. Here, we describe each of these activities in detail.

#### Activity 1: Level ground walking in an obstacle free environment

**Fig. 1:**
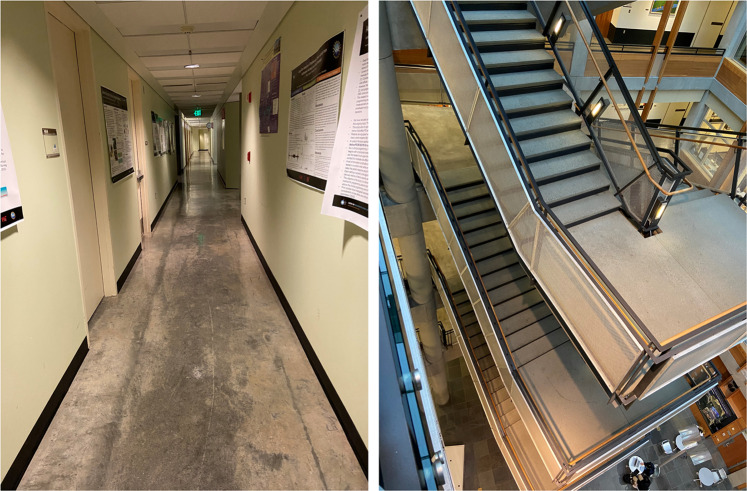
(left) Corridor for flat ground walking, and (right) Staircases spanning 6 floors.

This activity involved walking on flat ground in a long corridor (see Fig. 1) in the Paul Allen Building at University of Washington. Subjects were requested to walk along the corridor back and forth, at a self-selected pace. Each trial took approximately 2.5 minutes, with 4–5 rounds of the corridor. The duration of **data per subject** is 11.97 ± 6.66 minutes (mean ± std).

#### Activity 2: Level ground walking with random stops

This activity was performed in the same corridor as activity 1 (Fig. 1). For this activity, subjects followed instructions to stop and start walking at random. The instruction were provided using a voice recording, which was repeated across users. Subjects were not given any instructions on how fast to start or stop. The duration of **data per subject** is 13.55 ± 5.70 minutes (mean ± std).

#### Activity 3: Staircases spanning a 6-storey building

This activity consisted of stair ascent and descent in a 6-storey public building. This included sections of flat ground transitions in between levels (see Fig. 1). There are 13 consecutive stairs between flat sections and 26 stairs between 2 floors. The data was collected during regular work hours and there are other people using the stairs. The duration of **data per subject** is 11.52 ± 3.92 minutes (mean ± std).

#### Activity 4: Modified CHAMP

**Fig. 2:**
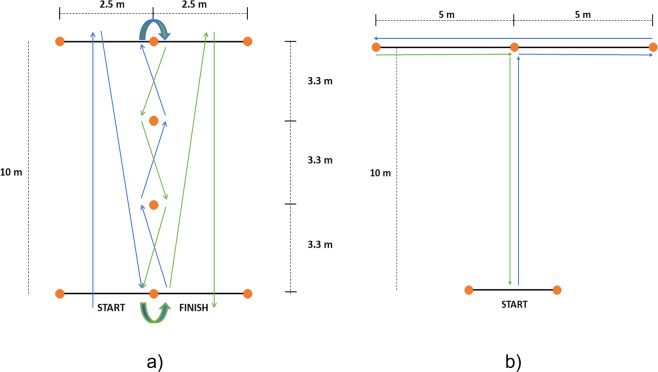
Modified CHAMP activities.
- Modified T-Test: Fig. 2b shows the activity course for the T-Test. Before beginning, subjects were asked to decide if they would first go left, or right. The subject started with walking forward for 10 meters, then stopped and started side-stepping to the right (or left) for 5 m, followed by side-stepping to the left (or right) for 10 m, and then returned to the middle cone. Lastly, they started backward walking, and stopped at the start position. The T-Test activity set consisted of 7 trials per subject. The duration of **data per subject** is 5.06 ± 0.49 minutes (mean ± std).

This data was collected as part of an experiment inspired from the standard Comprehensive High-Level Activity Mobility Predictor (CHAMP) test^25,26^, which is used to assess the agility of athletes and non-athletes. For the standard CHAMP test, the subject is asked to perform the task as fast as possible, and the time they take is used to assess their agility. We instructed the subjects to perform the task at a self-selected pace, with no requirement to finish the task as fast as possible. It is comprised of the following sub-activities:

- Modified Edgren Side-step Test: The subject started at rest with arm to the side. When cued by the experimenter, they side-stepped to the left for 10 seconds, stopped, and then side-stepped to the right for 10 seconds. This was repeated 3 times in each trial. The dataset includes 4 trials of the Edgren test per subject. The duration of **data per subject** is 4.48 ± 0.38 minutes (mean ± std).
- Modified Illinois Agility Test: The Illinois obstacle course is depicted in Fig. 2a. The subject began behind the start line with arms to the side and facing forward. When cued by the experimenter, the subject walked forward along the 10 m stretch, and then turned around and started walking towards the middle cone at the start line, then weaved up and down through the 4 center cones. Following this, they moved towards the center of 2.5 m line on the top-right side of the course, and touched the line. Lastly, they turned around and walked straight to the finish line. Five trials were collected from each subject. The duration of **data per subject** is 4.60 ± 0.41 minutes (mean ± std).

#### Activity 5: Controlled obstacle course

**Fig. 3:**
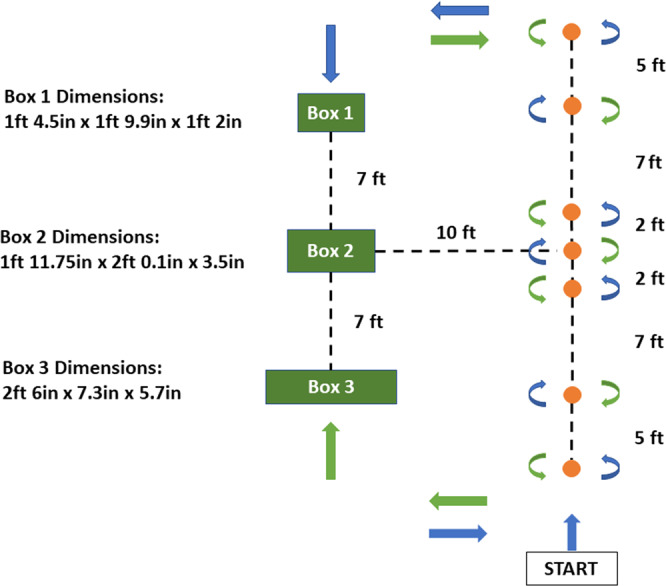
Controlled Obstacle Course: Cones in Orange; Boxes in Green. Box positions were randomly shuffled. Subjects avoid boxes by stepping over them.

The obstacle course is depicted in Fig. 3. The subject started with arms at rest to the sides. On ‘Go’ command, the subject started weaving up around the cones and was commanded at random to shift towards the left of the course and step over the boxes or to turn around and weave around the cones. Each trial consisted of the subject performing 5–7 rounds (some clockwise, some counter-clockwise) of the obstacle course. The duration of **data per subject** is 18.95 ± 4.54 minutes (mean ± std).

#### Activity 6: Unrestricted walking in public places

**Fig. 4:**
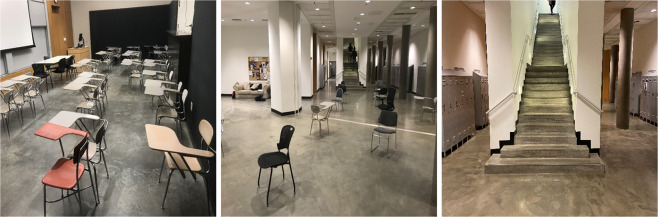
Environments: Classroom, Atrium and Staircases. Obstacles and other people using the spaces were not controlled.

**Fig. 5:**
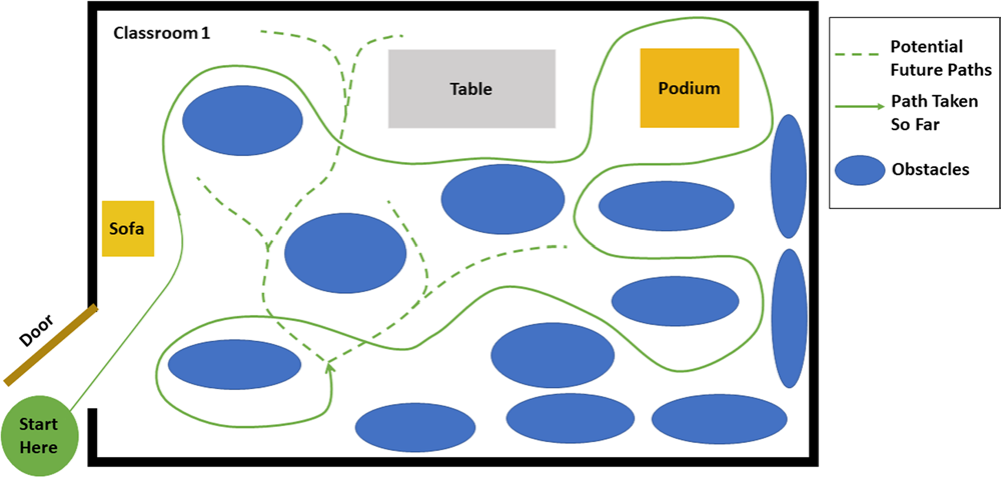
Classroom: Architecture of one of the classrooms. The arrangement of obstacles is not controlled and varies across subjects. The subject walks at self-selected speed and along self-selected path, The experimenter directed the subject to change their path only if the subject repeated the same path more than 2 times.

**Fig. 6:**
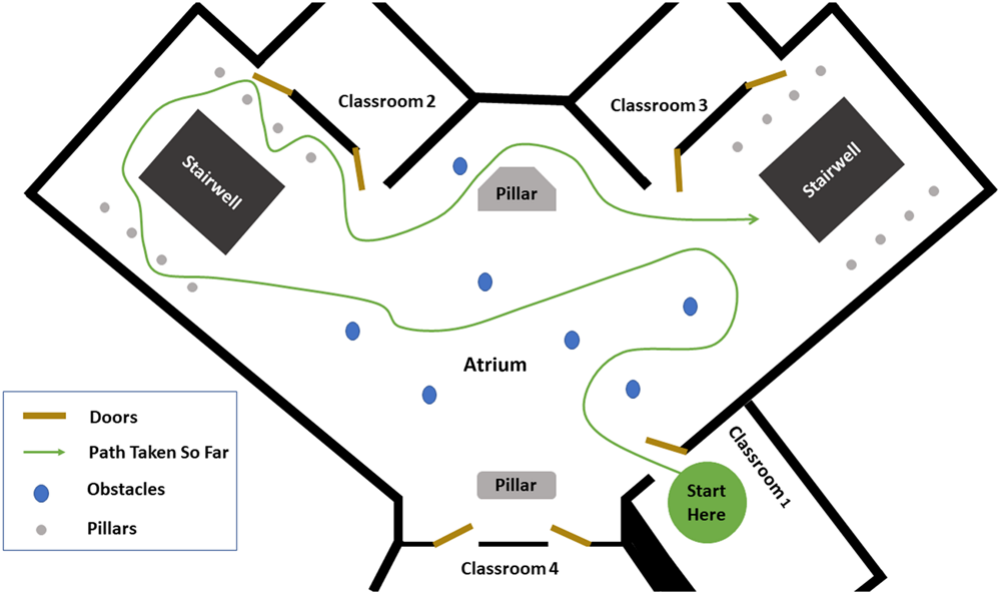
Atrium: Architecture of the Atrium. The arrangement of obstacles is not controlled and varies across subjects. The subject walks at self-selected speed and along self-selected path, The experimenter directed the subject to change their path only if the subject repeated the same path more than 2 times.

The environment was comprised of 3 main types of obstacle courses (Fig. 4): (1) Classrooms with dense obstacle arrangement, (2) An atrium with sparse obstacle arrangement, and (3) Two stairwells with a 33-step staircase each. The architectures for the classroom course and the atrium course are shown in Figs. 5, 6 respectively. The arrangement of obstacles was not controlled. Each trial consisted of the subject walking in each of the obstacle courses (except for subject xUD005, for whom there is no classroom data due to a scheduling conflict). An obstacle course from the three was randomly chosen and the subject was asked to go there and perform movements. Transitions while going from one obstacle course to another were also captured in the data. These included opening doors and flat ground to staircase transitions and vice-versa. Each subject’s movement speed and chosen path within an obstacle course was not constrained. Only which obstacle course to perform movements in was specified to them. If the subject started performing repetitive movements, e.g. always taking a left on encountering a particular obstacle or moving in a fixed loop several times, then they were instructed to vary their path by the experimenter. **data per subject** is 29.50 ± 5.40 minutes (mean ± std).

### Protocol

For each data collection session, the subject was briefed about the task to be performed and the equipment to be donned. Then, informed consent was obtained and documented. The participants then wore the Xsens suit and performed the standard n-pose calibration routine from Xsens. For activity 6 (unrestricted walking in public places), each subject also wore the pupil eye-tracker. Then, a pupil screen-marker calibration routine was performed, in which the subjects are instructed to gaze at several bullseye markers displayed on a laptop screen (https://docs.pupil-labs.com/core/software/pupil-capture/#calibration). Following this, the data collection began. The duration of each trial is described in the Tasks section above. Participants were encouraged to take breaks after each trial. The duration of the entire setup and data collection was around 1.5–2 hours.

### Data processing and synchronization

For activity 6, the vision and kinematics data was synced in post-processing using the timestamp data. Since the visual data frame rate is 30 Hz while the kinematics frame rate is 60 Hz, each vision frame was assigned a kinematics frame that was within 10 ms of it. The rest of the kinematics frames were dropped. All post-processing, including extraction of kinematics data from raw Xsens files and syncing with vision data, is done using a custom MATLAB script. Image frames from the videos were extracted using FFMPEG. Raw gaze coordinates are available in a csv file generated by the Pupil Player Software. These processes are described in more detail in the documentation provided with the data.

## Data Records

### Raw data

The raw data files are available on figshare^27,28^. The xsens motion capture files are available in ‘*.mvn’ file format. These files can be used for visualization using the MVN analyze software by Xsens^29^. This requires an Xsens license to be able to use the software. The corresponding xml files (‘*.mvnx’) are also available. These files contain the sensor position, segment orientation and joint angle data. An example MATLAB script is provided demonstrating how to read these raw data files. The corresponding raw eye-tracker data has also been uploaded. These files can be analyzed using the freely available Pupil Player software^30^.

### Processed data

**Fig. 7:**
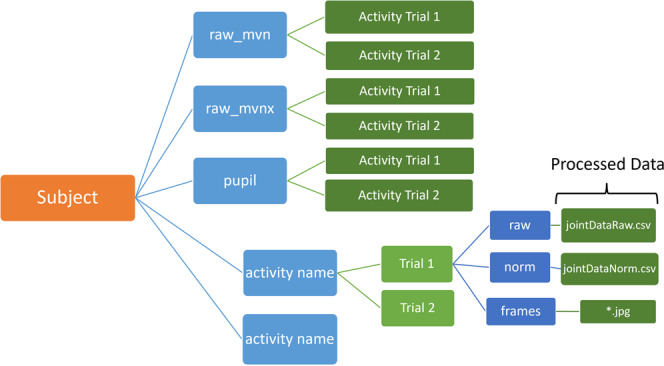
The data is stored in a hierarchical structure. Each leaf node (green, far right) is a file, stored in folders and subfolders (blue boxes) as depicted here.

In addition to the raw data files, we have also uploaded the processed motion capture and egocentric vision data. The kinematics data is available in csv file format. Each trial is stored in a separate csv file. The raw unnormalized joint data is stored in **‘jointDataRaw.csv’** (See Fig. 7). The min-max normalized joint data is stored in **‘jointDataNorm.csv’** (See Fig. 7). In a csv file, each row represents a single time instant. The columns store the corresponding timestamps, the names of the corresponding vision frames, and joint angle, joint velocity and acceleration data from the 66 joints. The python code for loading these files is available on (https://github.com/abs711/The-way-of-the-future). Vision frames are stored in the folder ‘frames’(See Fig. 7). The path to each vision frame is stored in the ‘jointDataRaw.csv’ and ‘jointDataNorm’ files.

### Data storage format

The dataset is structured in the format shown in Fig. 7. Each subject directory is comprised of raw data (mvn, mvnx, pupil) and processed files. The ‘raw_mvn’ folder contains all the trials for all the activities (named Activity-001). These files can be visualized using the MVN analyze software. The corresponding xml files are stored in the ‘raw_mvnx’ folder. The eyetracker and vision data is stored in the pupil folder. Each activity trial has a separate folder (named Activity-001), which can be dropped in the Pupil Player GUI (https://docs.pupil-labs.com/core/software/pupil-player/) to generate the egocentric video and the overlapped gaze. The processed kinematics data is stored in ‘.csv’ files, with jointDataRaw.csv containing the raw joint angle data and ‘jointDataNorm.csv’ containing the normalized data. The data is normalized using min-max scaling based on the joint-wise minimum and maximum values for the given trial. The processed vision data is stored in the frames folder in form of ‘.jpg’ images. Each row in the kinematics ‘.csv’ file, stores the name of the corresponding vision frame.

## Technical Validation

### Sensor placement and calibration

Subjects wore tight clothes provided by Xsens to prevent the slipping of IMUs. The sensors were placed as described in the Xsens Awinda documentation^31^ and the prescribed calibration procedure was followed. After the calibration, the body poses were verified manually in the MVN analyze software. This system has been validated using concurrent optical motion tracking and has been shown to be accurate, especially for sagittal plane movements^32^.

For the eye-tracker, the screen marker calibration procedure was followed as described in the Pupil documentation (https://docs.pupil-labs.com/core/software/pupil-capture/#calibration). The quality of calibration was verified manually by having the subject track the moving fingertip of the experimenter.

### Data synchronization

The data from the motion capture and the eye tracker was synchronized using the UNIX timestamps. The synchronization was validated by recording the hands of a subject while clapping. The instant the hands join was visualized in the MVN analyze visualization and the corresponding vision frame was also inspected. Another custom MATLAB script ‘custom_humanoid.m’ is available for verification on (https://github.com/abs711/The-way-of-the-future). The script generates a video which can be seen on the github repository.

## Usage Notes

This dataset can be used to reason about human movement, and especially how movement relates to the visual data arising from the environment. Here we will provide a brief overview of four studies we have conducted that use this dataset, as examples of the kinds of analysis that are possible. These notes will also serve as examples of how the data can be used in supervised vs. unsupervised ways, and with or without including the visual modality.

### Bootstrapping a visual classifier

**Fig. 8:**
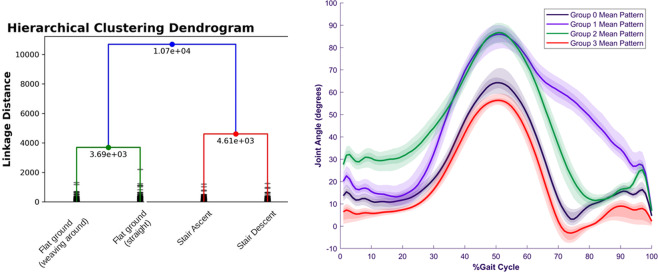
(left) Clustered gait types identified in^33^. Depending on how low in the hierarchy, the data could be divided into flat ground vs. stair walking, or further subdivided into turning gait, straight walking, and stair ascent and descent. (right) Mean knee angles for each of the clusters. The kinematic trajectories are distinct for different kinds of ambulation, leading to clusters that differentiate ambulation modes.

The first study^33^ is an approach for classifying ambulation modes, such as flat ground walking and stair ascent. Instead of defining these modes *a priori*, they are identified by clustering the movements of the knee joint. During different kinds of ambulation, the profile of knee movements is distinct. These clusters serve as pseudolabels of the terrain, requiring no human-annotated ground truth labels of terrain types. Figure 8 shows the clustered movement types and characteristic joint motions for each of the clusters.

The pseudolabels derived from kinematic clustering can then be used to train a supervised visual terrain classifier using the corresponding video data. The result is a visual terrain classifier with an accuracy of 96% that required no ground truth annotation. This kind of self-supervised bootstrapping is likely to become an important tool as datasets become increasingly large and large models become desirable to train. Example scripts for performing this analysis are available on Github in the directory named “Bootstrapping the visual classifier.” The file “gaitsteps_dataprep.m” performs gait segmentation in Matlab. The file “cluster_kinematics.py” performs the clustering of kinematics using sklearn libraries and saves the resultant labels as a pickle format. Finally, “cluster_classify_vision_train.py” and “cluster_vision_infer.py perform training and classification of images using the kinematic labels, based on transfer learning from the pretrained ResNet provided in the FastTorch library. These files may be used to reproduce the results^33^ and should serve as a basis for performing similar analysis.

### Optical flow for kinematics prediction

**Fig. 9:**
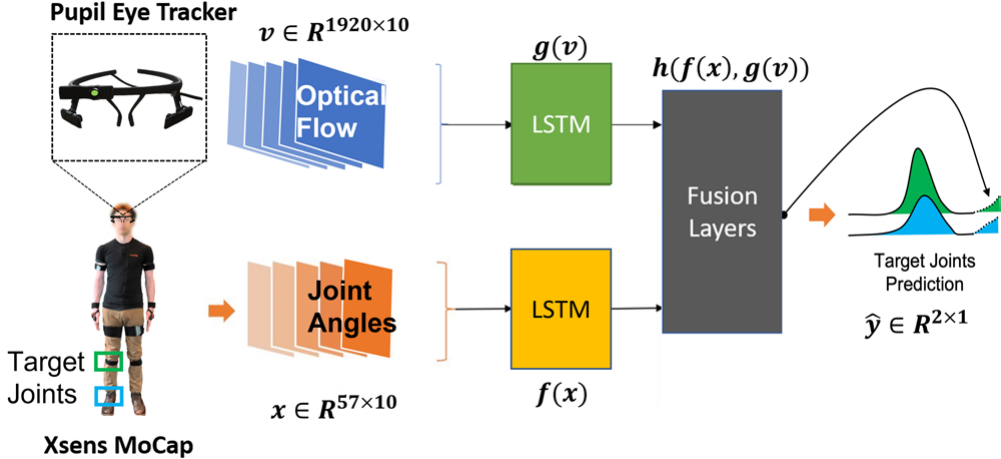
The neural network architecture combining vision and kinematics data^3^. The performance of this network (named as ‘Optical Flow’) is compared with the optical flow features zeroed out (named as ‘No Flow’). We find that using egocentric optical flow helps improve prediction of knee and ankle joint ankles from the rest of the body. The prediction performance is measured in terms of the root mean squared error (RMSE) between the predicted trajectory and the actual measured trajectory using motion capture.

The second example analysis is using either the kinematic data alone^2^ or also combining the the visual data^3^ to predict knee and ankle motion. See Fig. 9 for a depiction of how the data types are processed and combined to generate predictions. The idea here is that the movement of particular joints is highly predictable from a time history of the rest of the body motion and the visual scene. These predictions could be used as a reference trajectory for controlling an assistive wearable robot such as a prosthetic limb.

Figure 9 depicts the flow of data used in a regressor neural network. Visual data is processed into optical flow features, and then both sensory modalities are initially processed by a Long Short Term Memory (LSTM) neural network that models temporal dynamics. These two sensory streams are then fused in a network that predicts the target joint movements. “Optical Flow” refers to the full network and “No Flow” is an ablation case with the visual data zeroed out, to show its relative contribution. An interesting feature of this approach is that the system can target either instantaneous joint angles^2^ or can predict into the future^3^. We expect that predicting further into the future should be more difficult, but this has yet to be systematically studied to our knowledge.

**Fig. 10:**
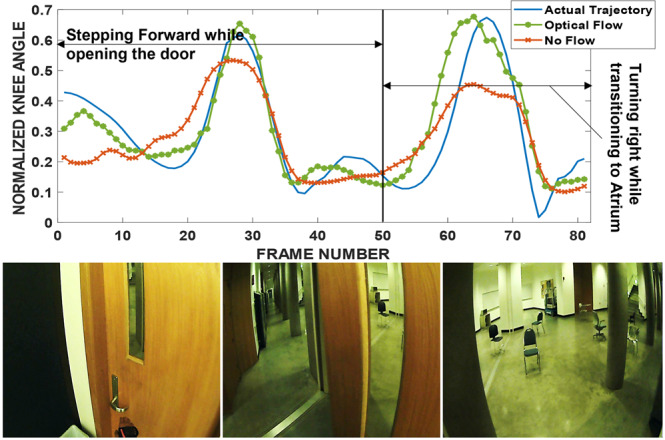
Gait kinematics (above) and the first, 40th, and 80th vision frames (below) during the maneuver. The subject exited the classroom and executed a right turn while entering the atrium. Optical Flow RMSE = 0.082, No Flow RMSE = 0.110.

**Fig. 11:**
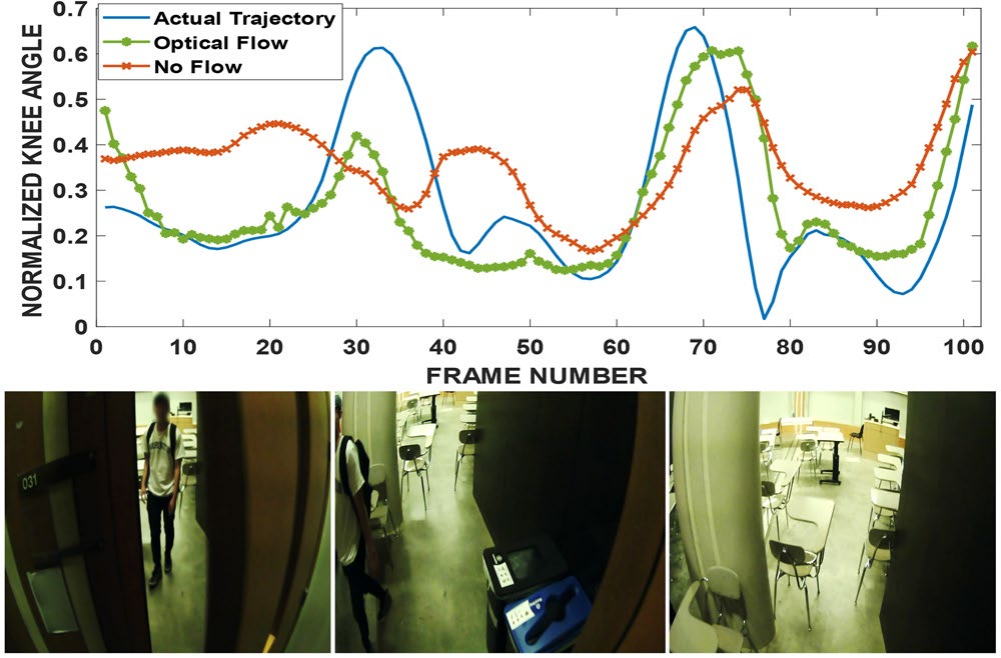
Frames number 15, 60 and 100 are shown. The subject opened the door and entered the classroom. Optical Flow RMSE = 0.131, No Flow RMSE = 0.188.

Figures 10, 11 show representative snippets of knee angle trajectories above, and three corresponding video frames. The actual measured trajectory of the participant (blue solid line) is somewhat estimable using only the kinematic measurements from the rest of the body (red square-dotted line). In general, however, additionally including the video data results in much better estimates (green circular-dotted line). More examples of this are provided in the manuscript^3^, including some examples of when visual data was not informative and did not improve predictions.

### Complexity of movement

**Fig. 12:**
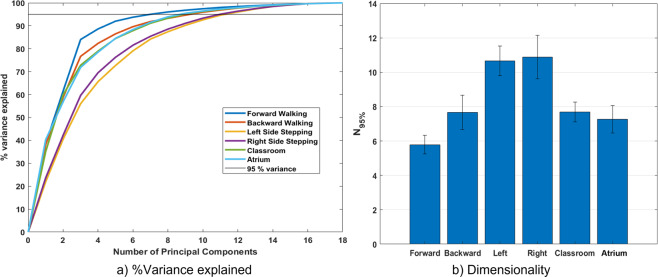
Commonly used measures of complexity provide quantitative insight into differences in activity type or behavior in and out of the laboratory. (**a**) depicts the percent variance explained by an increasing number of principal components, and (**b**) depicts the number of components necessary to represent 95% of the variability. These are both commonly used quantitative measures of complexity, yet they indicate, counterintuitively, that left and right sidestepping are more complex than obstacle avoidance or backward walking.

The third example^34^ is an analysis of the relative complexity of various locomotion activities. Forward walking, backward walking, sidestepping, and classroom walking were manually annotated using the Xsens Analyze visualizer. These annotations are used to organize the activities in the processed, but not the raw, form of the dataset files (see Fig. 7.) These activity types were compared in terms of their apparent complexity using a variety of measures from statistics, information theory and dynamical system theory. While not exhaustive, the study raised the important question of how should we measure the complexity of human locomotion, particularly when some of the standard complexity measures in the gait literature give counterintuitive results. For example, we expect locomotion in tight spaces with obstacles like a classroom to be more complex than obstacle free sidestepping. However, our analysis shows otherwise (See Fig. 12). A commmon measure of complexity, the number of principal components necessary to express 95 percent of the variability, rates left and right sidestepping as the most complex. This analysis is especially important considering that the data is measured from movement outside of an artificial laboratory environment. Although there has previously been some comparative analysis of treadmill walking and overground straight line walking, eg.^35^, this dataset provides the opportunity to analyze and compare more diverse activities outside of the lab.

### Dimensionality reduction and movement representations

**Fig. 13:**
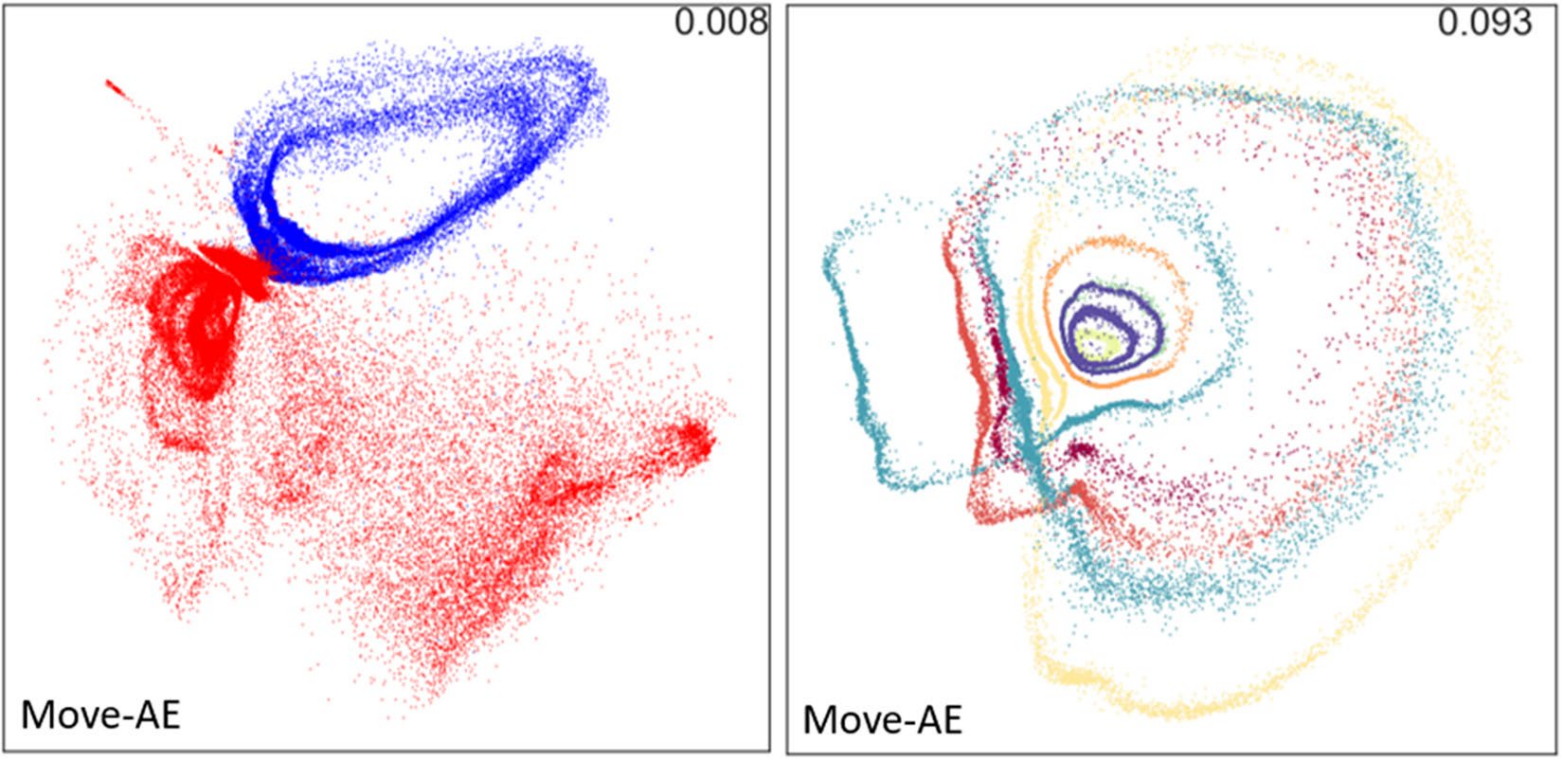
(left) Movements recorded from stair ambulation, in red, form a distinct cloud mostly separable from those recorded from flat ground walking, in blue. (right) Movements recorded from 8 individuals occupy distinct annular regions in the latent representation. In^36^ we show that a simple classifier can achieve 97% accuracy for distinguishing flat from stair walking, and 91% accuracy for discriminating amongst 8 individuals. These examples show that dimensionality reduction can assist in visualization, interpretation, or creating representations suitable for classification.

The final usage example is a means for representing the full-body kinematic data using a dimensionality reduction technique^36^. We compare principal component analysis (PCA) with two styles of autoencoding neural networks, dubbed Pose-AE and Move-AE. The Move-AE network uses recurrent neural network components to represent temporal dynamics, leading to higher Variance Accounted For (VAF) than the “snapshot” methods. We also show that the geometry of the low-dimensional latent space has human-interpretable structure, with different kinds of movement and different individuals occupying distinct regions (Fig. 13). This allows for simple classification of movement type and individual in the latent representations. Scripts demonstrating these techniques are made available in the directory named “Dimensionality reduction and movement representation”.

## Data Availability

We provide an example python script for loading the processed motion capture and vision data, named ‘main.py’ in the directory ‘data_loading_example’ on the Github repository. In addition we provide scripts for synchronization and frame-dropping, and examples of loading into pytorch machine learning pipeline. All code is available on (https://github.com/abs711/The-way-of-the-future).
